# Supplementary material for: Epstein–Barr virus induces aberrant B cell migration and diapedesis via FAK-dependent chemotaxis pathways
Source: Nat Commun. 2025 May 19;16:4581. doi: 10.1038/s41467-025-59813-z (PMC12089463; doi:10.1038/s41467-025-59813-z)
Supplement: Supplementary file 1 — Supplementary Information [file 41467_2025_59813_MOESM1_ESM.pdf]

Epstein-Barr virus induces aberrant B cell migration and diapedesis via FAK-dependent chemotaxis pathways

First author: Susanne Delecluse[1,2,3,4],

Corresponding author: Henri-Jacques Delecluse[1,2,*]

[1]Unit D400, DKFZ, Heidelberg, Germany

[2]Inserm joint unit, Heidelberg, Germany

[3]Department Nephrology, University of Heidelberg, Germany

[4]German Center for Infection Research (DZIF), Braunschweig, Germany.

* corresponding author, h.delecluse@dkfz.de

Supplement

Supplementary Figure 1

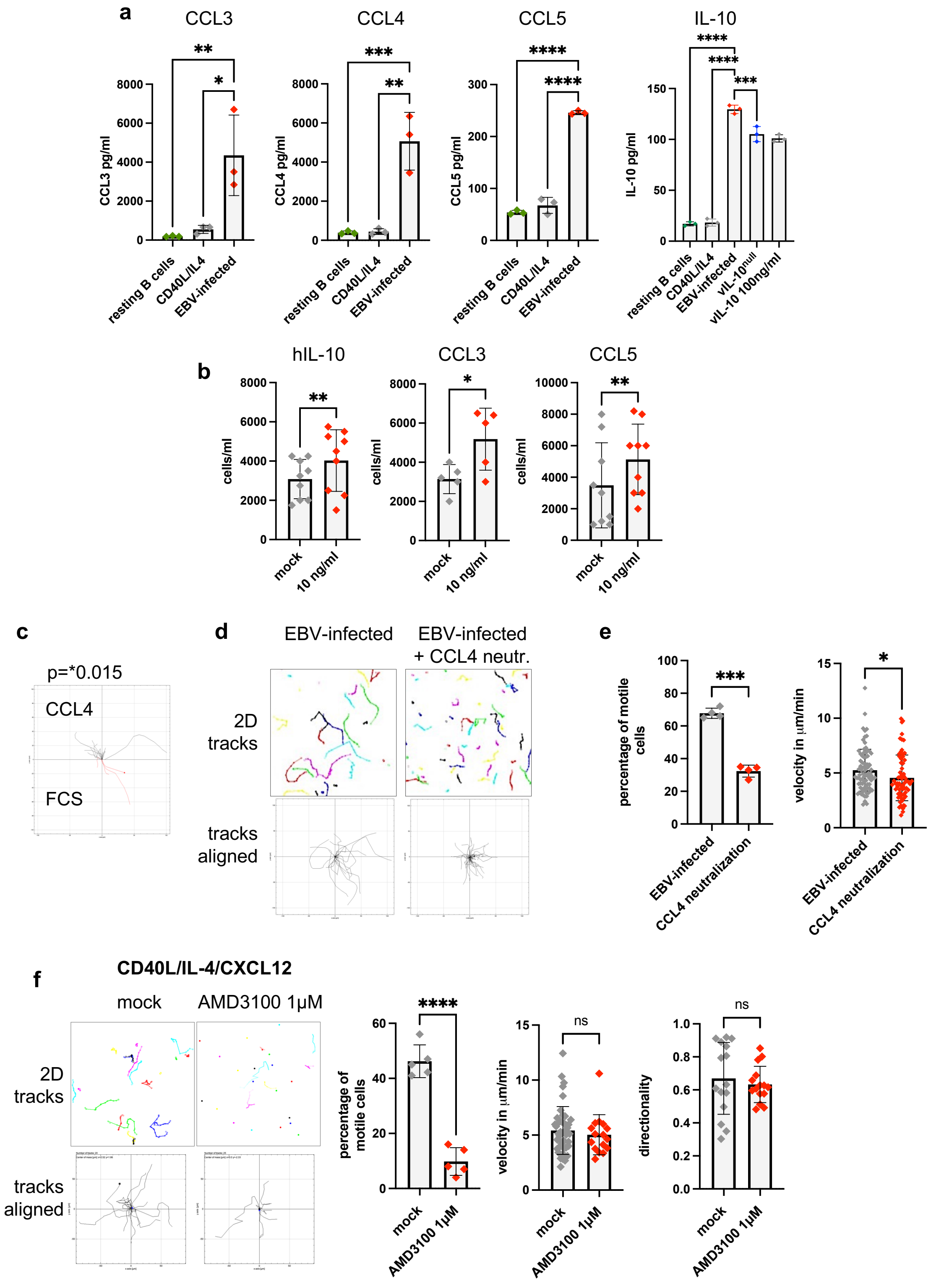

Suppl. Fig. 1 CCL3, CCL4, CCL5 attract EBV-infected B cells. a) Concentrations of CCL3, CCL4, CCL5 and IL-10 in supernatants of resting primary B cells, B cells stimulated with CD40L+IL-4 and EBV-transformed B cells were determined by ELISA. The latter included cells transformed by wild type EBV or by an EBV-encoded IL-10 knockout (vIL-10^{null}).

Purified EBV-encoded IL-10 served as a positive control. Bar graphs show mean and standard error of the mean (n=3 independent B cell samples). b) Transwell assays with human IL-10 (hIL-10), CCL3, or CCL5 as attractors. 5 x 10E4 cells were seeded in the top chamber. Medium containing one of the chemokines (10 ng/ml) was placed in the bottom chamber. The number of cells that have reached the bottom chamber after 1h is given in bar graphs as mean with standard deviation (n=9, including three individual experiments performed in triplicate with 3 independent B cell samples). Medium devoid of chemokines served as a negative control. c) CCL4 attracts EBV-transformed B cells. 100 ng of CCL4 was placed at one extremity of a collagen matrix containing EBV-transformed B cells. The paths of cells migrating in the direction of the gradient or in its opposite direction are given. Statistical analysis using Rayleigh test showed preferential migration towards CCL4. d) Neutralizing antibodies against CCL4 reduce migration of EBV-infected B cells. The paths of EBV-infected B cells seeded at high density in a collagen matrix in the presence or absence of neutralizing antibodies (10 µg/ml) against CCL4 were recorded by live cell imaging over 15 minutes and given as 2D tracks in their native conformation (Top) or centered at the origin (bottom) (n=4 independent B cell samples). e) Analysis of the tracks generated in d) determined the percentage of mobile cells and their velocity (n=20, from 4 experiments with 4 independent B cell samples). Bar graphs give the mean and standard deviation. f) The CXCR4 inhibitor AMD3100 suppresses migration of blasts stimulated with CD40L, IL-4 and CXCL12. The paths of B blasts seeded at high density in a collagen matrix in the presence or absence of AMD3100 (1 µM) were recorded by live cell imaging over 15 minutes and given as 2D tracks in their native conformation (top) or centered at the origin (bottom). The percentage of motile cells is indicated in the bar graph (5 experiments with independent B cell samples). Velocity and directionality were determined for motile cells ((n=15, from 5 experiments with 5 independent B cell samples). Bar graphs give means with standard deviations. Statistical analysis was done using one way analysis of variance in a) and two-sided paired t-tests in b), e) (percentage of motile cells) and f) (percentage of motile cells) and two-sided unpaired t-test in e) and f) for velocity and directionality * = $p < 0.05$, ** = $p<0.01$, *** = $p<0.001$ and **** = $p<0.0001$. Source data are provided as a Source Data file.

Supplementary Figure 2

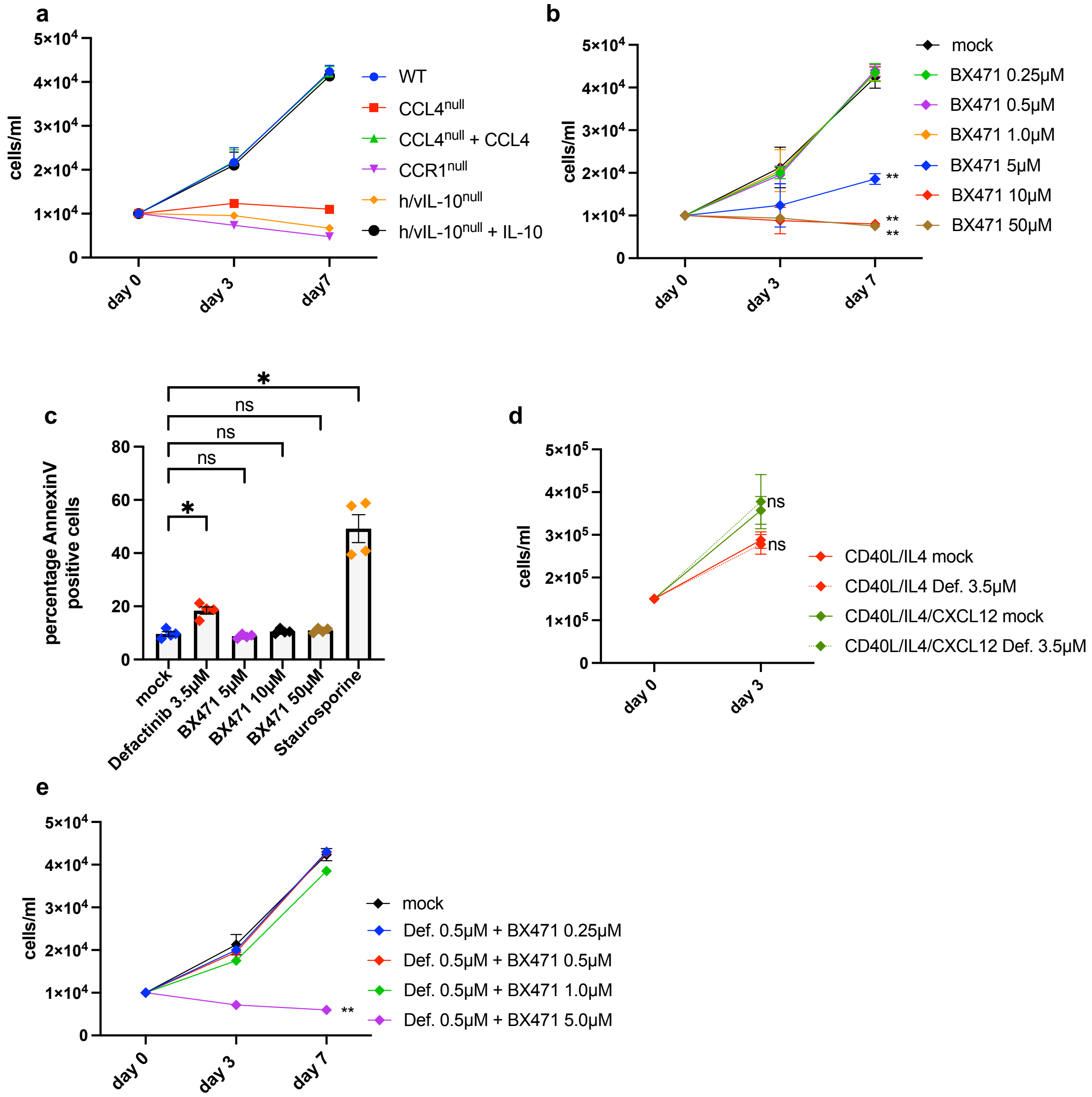

Suppl. Fig. 2 B cell growth is controlled by CCR1, CCL4 and IL-10. a) The growth of B cells transformed by various EBV mutants was monitored over 7 days. Cell lines studied included a $CCR1^{null}$, a $CCL4^{null}$ and an EBV-encoded and human double $IL\text{-}10^{null}$ mutant ($h/vIL\text{-}10^{null}$). B cells transformed by the $CCL4^{null}$ and the $h/vIL\text{-}10^{null}$ mutants were grown in the presence or absence of CCL4 and IL-10. B cells transformed by wild type EBV served as a positive control. Mean and standard deviation is given for indicated time points (n=5 independent B cell samples). b) Wild type EBV-transformed B cells were grown for 7 days in the presence of BX471, a CCR1 inhibitor, at various concentrations. Mean and standard deviation is given for indicated time points (n=5 independent B cell samples). c) EBV-infected B cells treated with the BX471 CCR1 inhibitor or with the FAK2 inhibitor defactinib for 24 hours were stained for annexin V surface expression by flow cytometry. Cells exposed to Staurosporin served as a positive control. Bar graphs give the mean and standard deviation (n=4 independent B cell samples). d) B cells stimulated with CD40L and Il-4 or with CD40L, Il-4 and CXCL12 were exposed to defactinib (3.5 µM). Cell growth was monitored over 3 days. Mean and standard deviation is given for indicated time points (n=5 independent B cell samples). e) EBV-infected B cells were treated with a combination of defactinib and BX471 at

various concentrations. Mean and standard deviation are given for the indicated time points (n=5 independent B cell samples). Statistical significance was determined using paired t-tests for values at day 3 in d), or 7 in a), b) and e) and one-way analysis of variance in c). ** = $p<0.01$. Source data are provided as a Source Data file.

Supplementary Figure 3

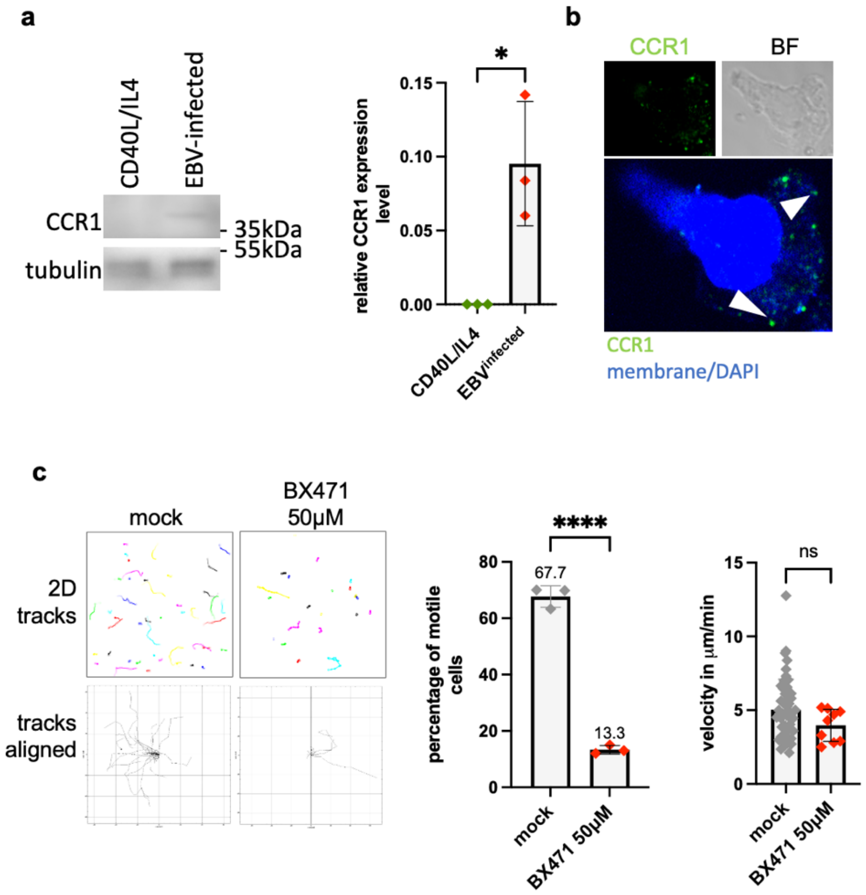

Suppl. Fig. 3 CCR1 expression and function in EBV-infected B cells. a)Western blot analysis of B cells infected by EBV or stimulated with CD40L/IL-4 using a CCR1-specific antibody. The bar graph summarizes the results obtained with three independent primary B cell samples. Mean and standard deviation are given. b) Immunofluorescence studies on EBV-infected B cells using a CCR1-specific antibody. The arrows show the cell lamellipodia (n=30, one representative example from 3 experiments with independent B cell samples). c) The paths of EBV-infected B cells seeded at high density in a collagen matrix in the presence or absence of the CCR1 inhibitor BX471 at 50 μM were recorded by live cell imaging over 15 minutes and given as 2D tracks in their native conformation (top) or centered at the origin (bottom). Analysis of the generated tracks determined the percentage of mobile cells and their velocity (n=3 independent B cell samples). Bar graphs give the mean and standard deviation. d) EBV-negative Burkitt cell lymphoma BL41 cells were transfected with expression plasmids expressing LMP1 or EBNA2 or with a vector control. The percentage of cells expressing CCR1 was determined by FACS. CCL4 concentrations in supernatants of these cells were determined by ELISA. Results are given in pg/ml. Bar graphs show mean CCR1 or CCL4 expression with standard deviation (n=3 independent transfections). Statistical significance was determined using two-sided paired in a) and c) (motility) and unpaired t-tests in c) (velocity) and one-way analysis of variance in d). **** = $p<0.0001$. Source data are provided as a Source Data file.

Supplementary Figure 4

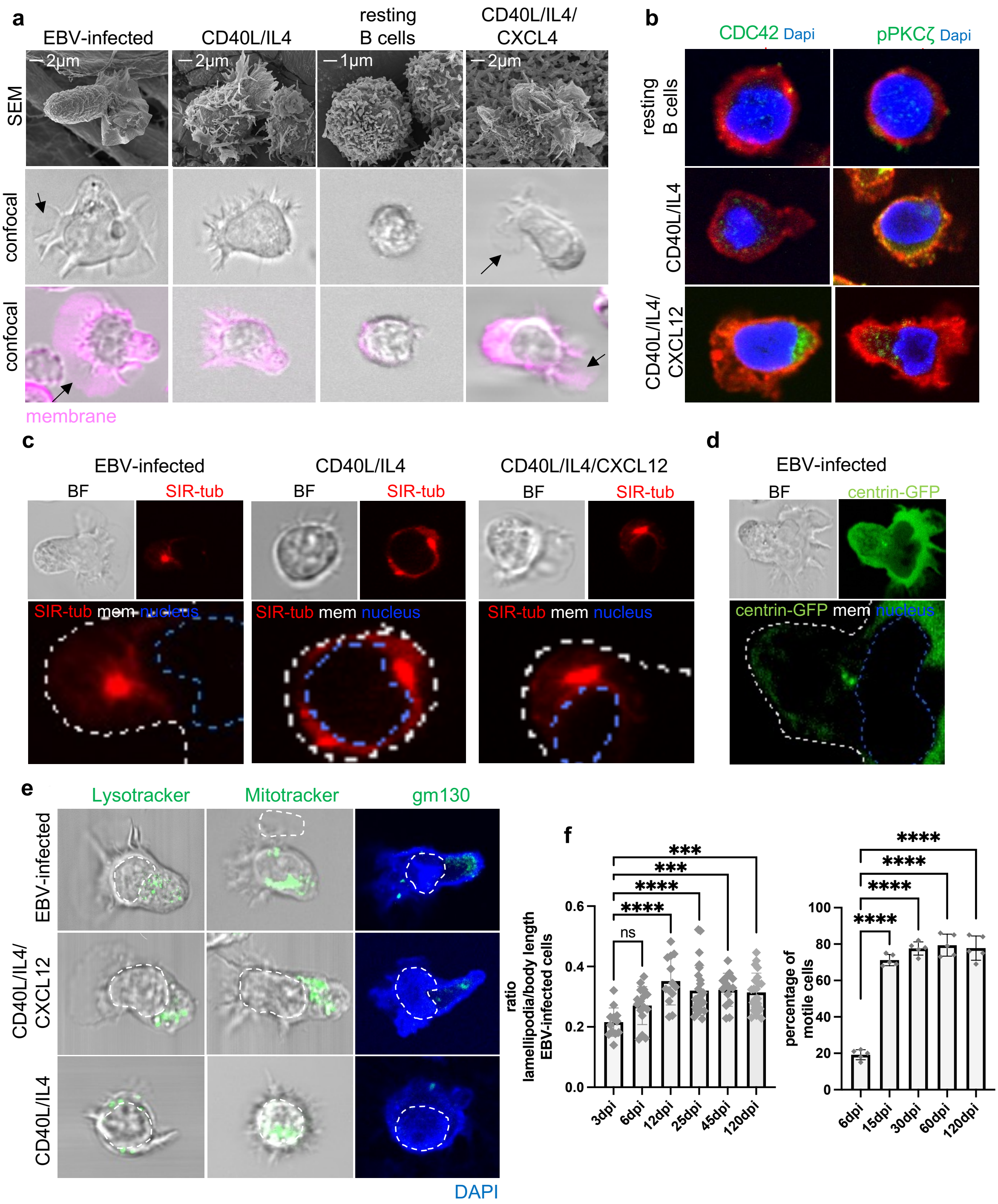

Suppl. Fig. 4 EBV-infected B cells are polarized (I). a) EBV-infected B cells, primary B cells stimulated with CD40L/IL-4, resting primary B cells, and primary B cells stimulated with CD40L/IL-4 and CXCL12 were subjected to scanning electron microscopy or confocal microscopy. Cells were also stained with a membrane dye (purple) and investigated by conventional microscopy (n=30, one representative example from 3 experiments with independent B cell samples). Arrows indicate lamellipodiae. b) Resting B cells, primary B cells stimulated with CD40L/IL-4 and primary B cells stimulated with CD40L/IL-4 and CXCL12 were stained with antibodies specific for CDC42 and phosphoPKCzeta (both green), DAPI staining shows the cell nuclei (n=30, one representative example from 3 experiments with

independent B cell samples). c) EBV-infected B cells, primary B cells stimulated with CD40L/IL-4 and primary B cells stimulated with CD40L/IL-4 and CXCL12 were stained with a SIR-tubulin probe to highlight their tubulin network and identify the microtubule organizing center (MTOC). The uropode is delineated with a white dashed line, the nucleus with a blue dashed line (n=50, one representative example from 3 experiments with independent B cell samples). d) EBV-infected B cells were transfected by a plasmid that encodes a centrin-GFP fusion protein. This allows detection and localization of the centrioles in these cells. The uropode is delineated with a white dashed line, the nucleus with a blue dashed line (n=50 one representative example from 3 experiments with independent B cell samples). e) EBV-infected B cells, primary B cells stimulated with CD40L/IL-4 and CXCL12, and primary B cells stimulated with CD40L/IL-4 were stained with lysotracker or mitotracker to localize lysosomes and mitochondria in live cells. The analysis was completed by an immunostain using antibodies specific for the Golgi apparatus protein gm130 (n=50 one representative example from 3 experiments with independent B cell samples). White dashed lines surround cell nuclei. f) The development of the lamellipodial appendage in EBV-transformed B cells and their migrating abilities were monitored for 120 days after EBV infection. Both the ratio between the size of the lamellipodia and the size of the main cell body (left panel, n=25 one representative example from 3 experiments with independent B cell samples) and the percentage of migrating cells (right panel, n=4 independent B cell samples) are indicated Bar graphs give the mean and standard deviation. Statistical significance was determined using one-way analysis of variance. *** = $p<0.001$ and **** = $p<0.0001$. Source data are provided as a Source Data file.

Supplementary Figure 5

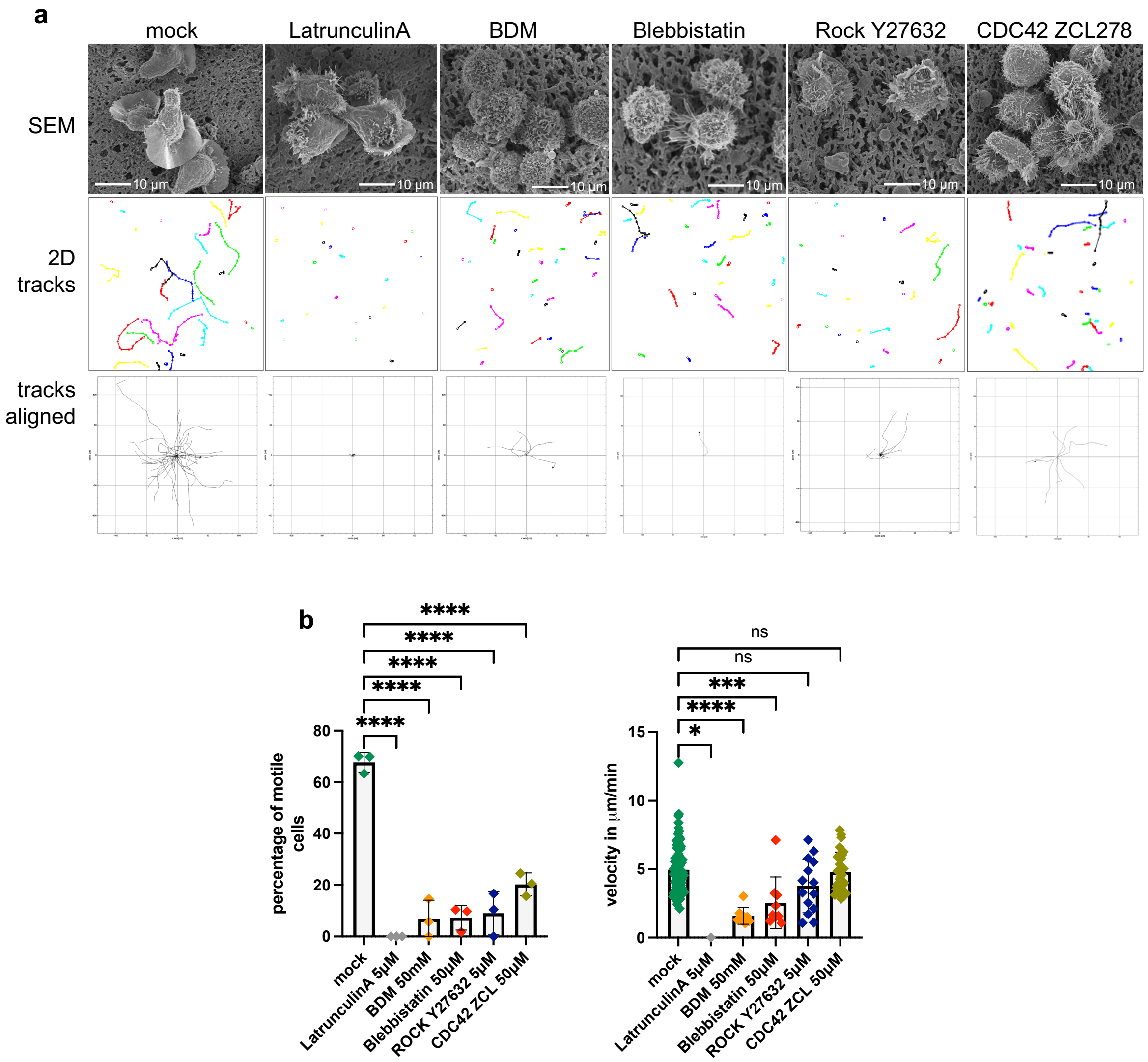

Suppl. Fig. 5 Polarization and migration of EBV-infected B cells requires actin and myosin polymerization and is controlled by CDC42 and ROCK. a) EBV-infected B cells were treated with F-actin (Latrunculin A 5µM) and myosin inhibitors (BDM 50 mM and Blebbistatin 50 µM) for 15 minutes. Infected B cells were also exposed to the ROCK inhibitor Y27632 (5 µM) and the CDC42 inhibitor ZCL278 (50 µM). The morphology of the treated cells was determined using scanning electron microscopy (n=30, one representative example from 3 experiments with independent B cell samples). Treated cells were also seeded in a collagen matrix and their migration was recorded by live cell imaging over 15 minutes. The 2D tracks are shown in their original (top) or centered at the origin (bottom) conformations. b) The graph shows the percentage of motile cells and their velocity after treatment (n=3 independent B cell samples). Bar graphs represent the mean and standard deviation. Statistical significance was determined using one-way analysis of variance. * = p <0.05, *** = p<0.001 and **** = p<0.0001. Source data are provided as a Source Data file.

Supplementary Figure 6

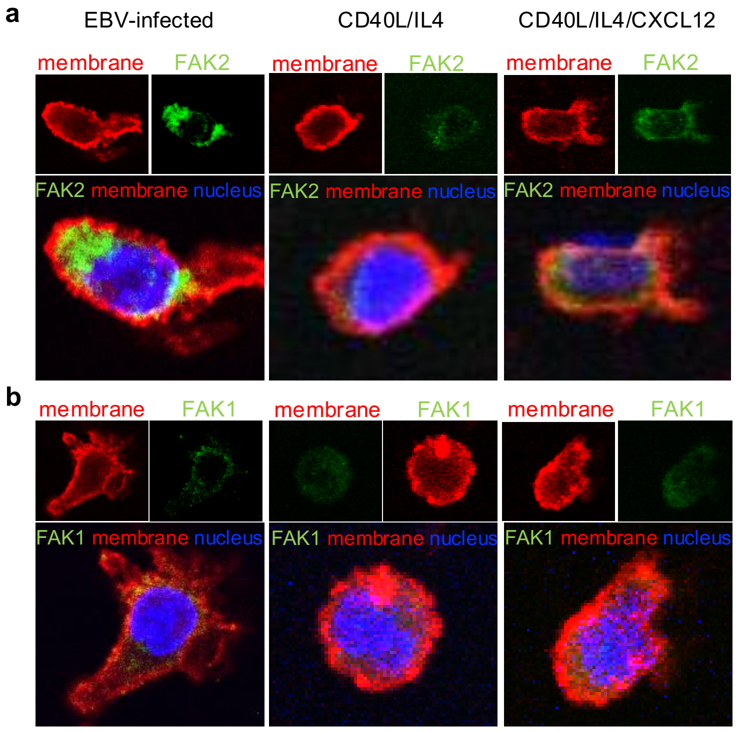

Suppl. Fig. 6 EBV-infected B cells express FAK2 but not FAK1 at high level. a) Immunofluorescence studies on EBV-infected B cells, primary B cells stimulated with CD40L/IL-4 and primary B cells stimulated with CD40L/IL-4 and CXCL12 using a FAK2-specific antibody. Cells were counterstained with a red membrane dye and with DAPI to stain the cell nuclei (n=30, one representative example from 3 experiments with independent B cell samples). b) Same as in a) with a FAK1-specific antibody (n=30, one representative example from 3 experiments with independent B cell samples).

Supplementary Figure 7

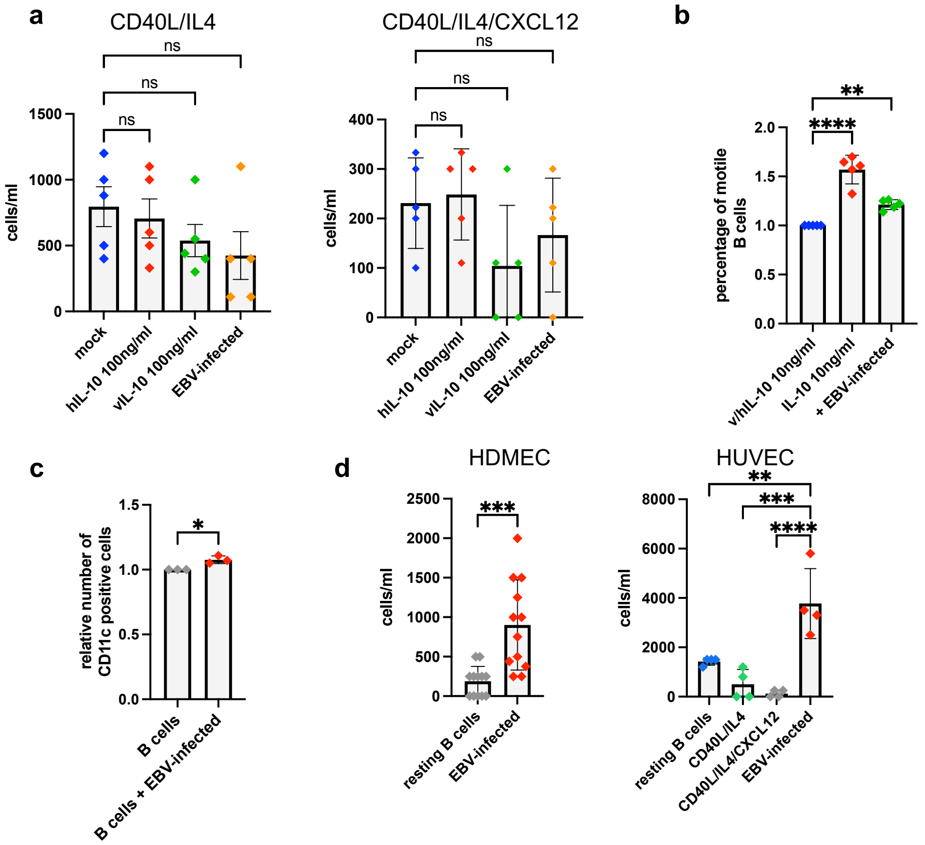

Suppl. Fig. 7 IL-10 increases motility of primary B cells. a) Primary B cells stimulated with CD40L/IL-4 and primary B cells stimulated with CD40L/IL-4 and CXCL12 were subjected to a chemotaxis assay with human (hIL-10) and EBV-encoded IL-10 (vIL-10), as well as with EBV-infected B cells. None of these conditions increased cell migration above control levels. Bar graphs give the mean and standard deviation (n=5 independent B cell samples). b) Primary B cells exposed to a mix of EBV-encoded and human IL-10 (10 ng/ml each) or cocultivated with EBV-infected B cells were seeded in collagen and subjected to time lapse microscopy. The bar graphs indicate the percentage of motile cells, relative to untreated cells (n=5 independent B cell samples). c) Primary B cells were co-cultivated with EBV-infected B cells for 24 hours and stained for CD11c by FACS to determine the proportion of CD11c+ cells in the population before and after coculture (n=3 independent B cell samples, 2000 cells recorded). The effects of the coculture on the proportion of CD11c+ after coculture are given relative to the CD11c+ proportion before coculture. Bar graphs give the mean (1.06 for the co-culture with EBV-infected B cells) and standard deviation. d) Spontaneous diapedesis of primary resting B cells and of EBV-infected B cells through a layer of HDMEC or HUVEC. Bar graphs give the mean and standard deviation of cell numbers that had migrated after 24h of culture (n=12, 4 individual experiments preformed in triplicate). Statistical significance was determined using one-way analysis of variance in a), b) and d) (HUVEC) and using a two-sided paired t-test in c) and d) (HDMEC). ** = $p<0.01$, *** = $p<0.001$ and **** = $p<0.0001$. Source data are provided as a Source Data file.

Supplementary Figure 8

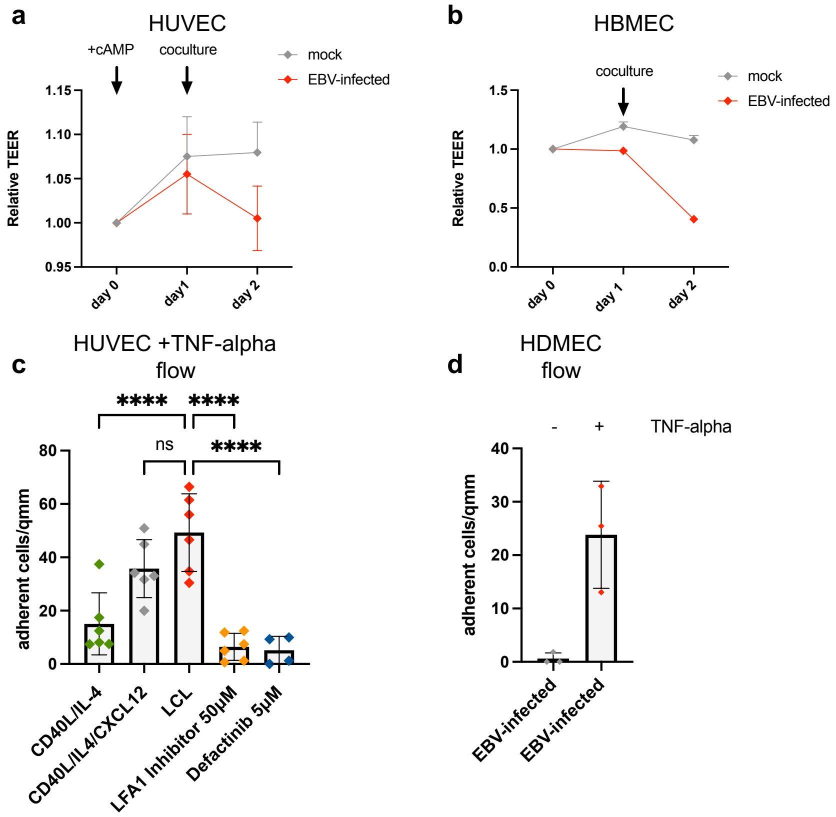

Suppl. Fig. 8 EBV-infected B cells modify the permeability of endothelial cell barriers. a) HUVEC cells were induced to form a tight endothelial barrier with cAMP. One day later, they were incubated with EBV-infected B cells. TEER was measured at each time point. Untreated HUVEC cells served as a negative control. Mean and standard deviation is given for indicated time points as ratio to the values recorded on day 0 (n=5 independent B cell samples). b) HBMEC cells were investigated as in a), except that they do not need to be induced. Untreated HBMEC cells served as a negative control. Mean and standard deviation is given for indicated time points as ratio to the values recorded on day 0 (n=5). c) The graphs show the concentration of various type of B cells adhering to HUVEC activated with TNF-α under constant flow conditions. The ability of EBV-infected B cells to bind to HUVEC after treatment with LFA1 inhibitor BIRT377 50 µM or FAK2 inhibitor defactinib 3.5 µM is also given. Mean and standard deviation is shown (n=5 independent B cell samples). Statistical significance was determined using one-way analysis of variance. d) The graphs show the concentration of EBV-infected B cells adhering to HDMEC cells under constant flow conditions, with or without prior TNF-α stimulation (n=3 independent B cell samples). Bar graphs give the mean and standard deviation. **** = p<0.0001. Source data are provided as a Source Data file.

Supplementary Figure 9

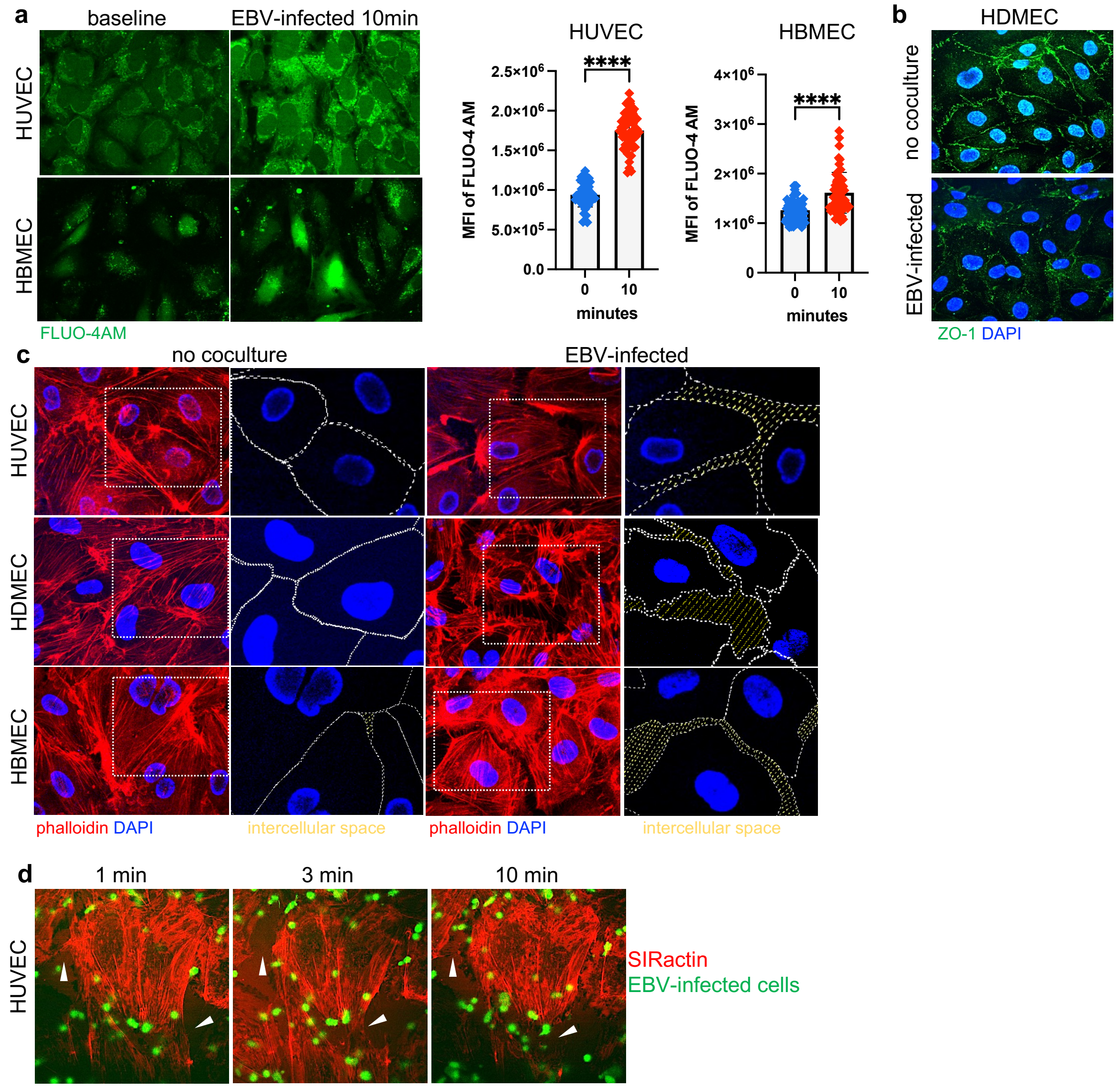

Suppl. Fig. 9 EBV-infected B cells profoundly modify the architecture of endothelial cell barriers. a) HUVEC and HBMEC cells were co-cultivated with EBV-infected B cells for 10 minutes. Endothelial cells were stained with Fluo-4AM and fluorescence signals were recorded with a confocal microscope and quantified with Image J. The results of the analysis are given in a bar graphs showing mean and standard deviation. Statistical significance was determined using two-sided unpaired t-test (n=30, one representative example from 3 experiments with independent B cell samples). b) EBV-infected B cells were cocultivated with HDMEC. Endothelial cells were stained with an antibody specific for ZO-1 and counterstained with DAPI. c) HUVEC, HDMEC and HBMEC layers were co-cultivated with EBV-infected B cells for 30 minutes. Cells were stained with SIRactin 24h later. Intercellular spaces are shown as yellow dashed lines (n=50, one representative example from 3 experiments with independent B cell samples). d) HUVEC layers cells were stained with SIRactin, co-cultivated with stained EBV-infected B cells (green) and subjected to time lapse microscopy. Pictures were recorded one, three and 10 minutes after the beginning of the coculture. The arrows show the increasing

defects in the actin structure of the same cell (n=30, one representative example from 3 experiments with independent B cell samples). **** = $p<0.0001$. Source data are provided as a Source Data file.

Supplementary Figure 10

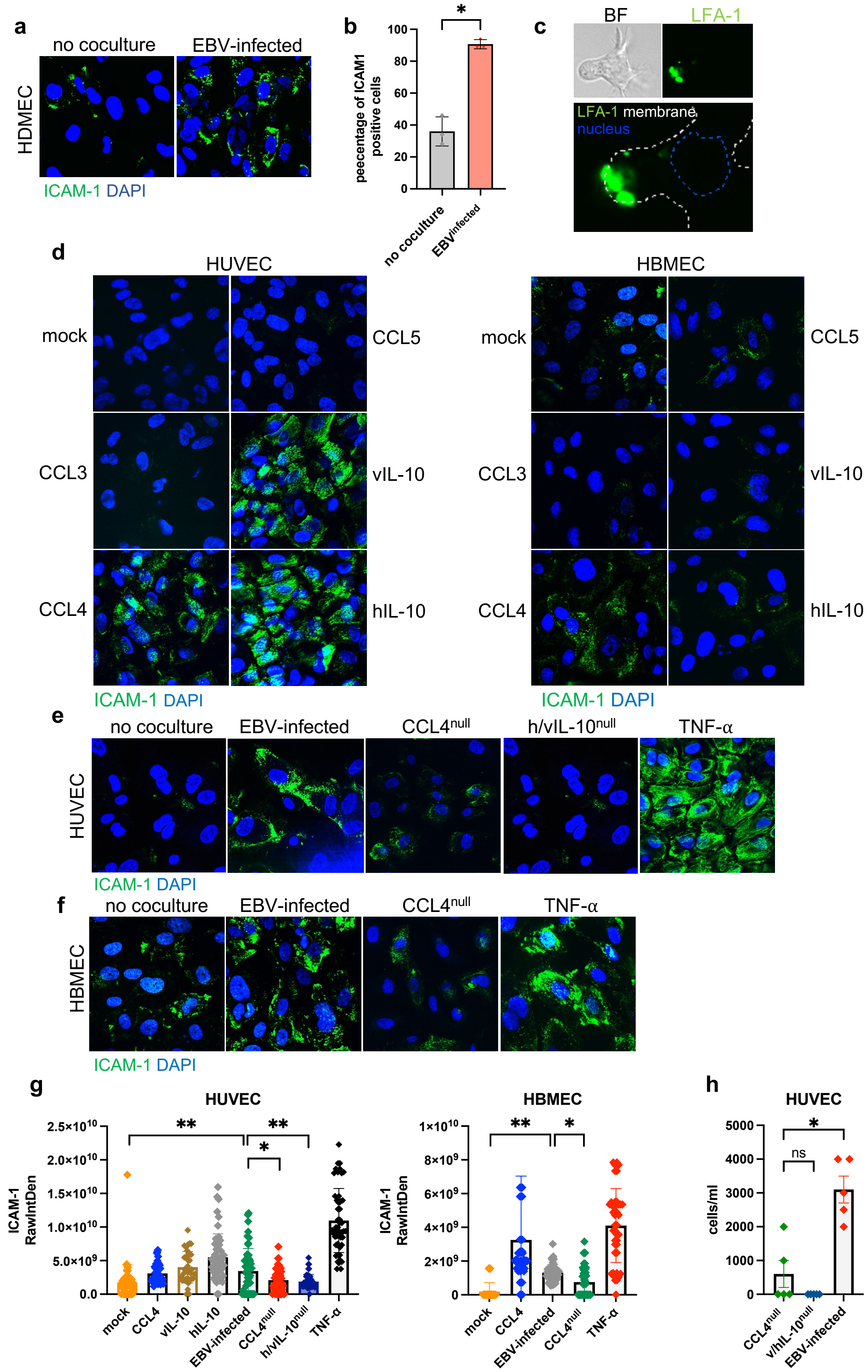

Suppl. Fig. 10 EBV-infected B cells activate ICAM-1 expression in endothelial cells. a) HDMEC endothelial cells were cocultured with EBV-infected B cells for 30 minutes. The effect of coculture on ICAM-1 expression was evaluated 24 hours later by immunofluorescence (n=50, one representative example from 3 experiments with independent B cell samples). b) The graph shows the percentage of ICAM-1 positive cells before and after coculture as defined

by FACS in HBMEC cells (n=3 independent B cell samples, 2000 cells recorded). Bar graphs give the mean and standard deviation. Statistical significance was determined using two-sided paired t-test. c) Immunofluorescence staining shows LFA-1 expression at the uropode of EBV-infected B cells (n=50, one representative example from 3 experiments with independent B cell samples). d) HUVEC and HBMEC cell layers were treated with CCL3, CCL4, CCL5, human (hIL-10) and EBV-encoded IL-10 (vIL-10) for 24 hours (all cytokines at 100ng/ml) and stained for ICAM-1 expression (n=50, one representative example from 3 experiments). e) HUVEC layers were subjected to co-culture with various types of EBV-infected B cells (wild type, $CCL4^{null}$, h/vIL-10^{null}) for 24 hours and stained for ICAM-1 expression (n=50, one representative example from 3 experiments with independent B cell samples). HUVEC treated with TNF-α served as a positive control. f) same as in e) for HBMEC co-cultured with wild type or $CCL4^{null}$ infected B cells. g) Bar graphs summarizing the results obtained in d), e) and f). Results are given as raw integrated density of fluorescence (Raw/Int/Den). (h) Diapedesis of wild type, $CCL4^{null}$, h/vIL-10^{null} infected B cells through a HUVEC layer. Bar graphs give the mean and standard deviation. Statistical significance was determined using one-way analysis of variance in g) and h). * = p <0.05 , ** = p<0.01. Source data are provided as a Source Data file.

Supplementary Figure 11

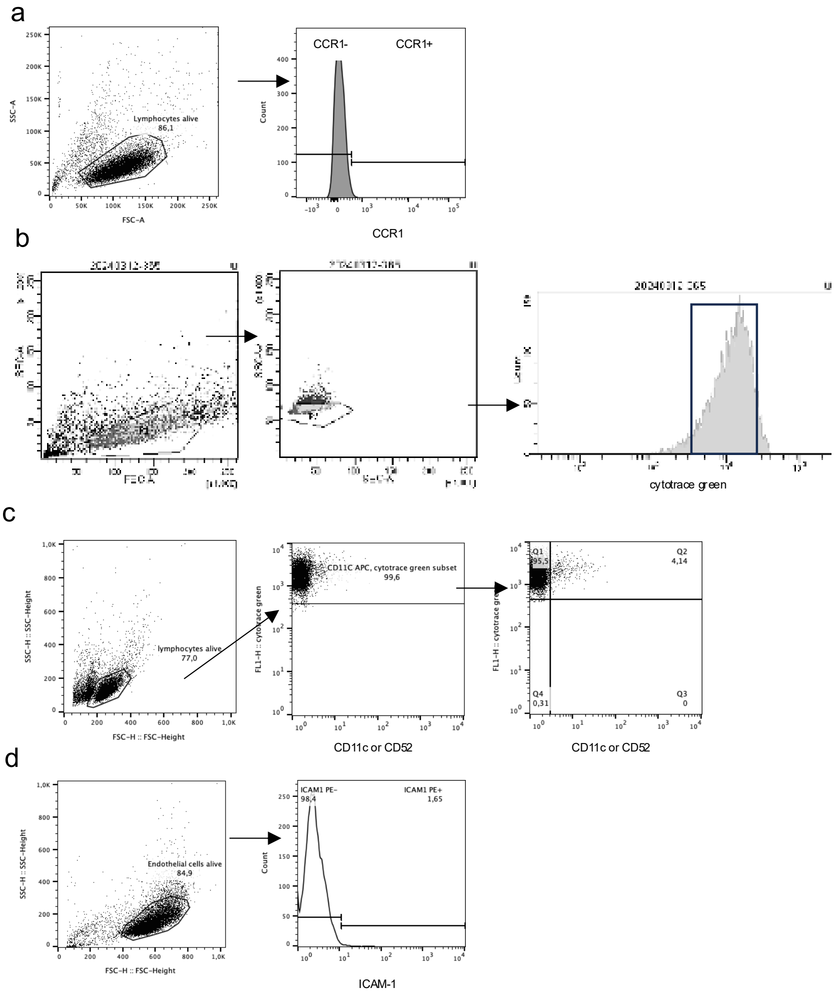

Suppl. Fig. 11 Gating strategies for flow cytometry analyses. a) Gating strategy to determine percentage of cells expressing CCR1 (Fig. 2e-g and Supp. Fig. 3d). b) Gating strategy for single cell sorting and subsequent scRNA analyses (Fig. 4e-f). c) Gating strategy to determine percentage of cells expressing CD52 (Fig. 4f) or CD11c (Fig. 4g and Fig. 5f and Supp. Fig. 7c). d) Gating strategy to determine percentage of cells expressing ICAM-1 (Supp. Fig. 10d).

Supplementary Tables

**usage**	Fwd: 5´- TCGAGTAGTAGAACTTCTGG - 3´<br>Rev: 5´- AATTCCAGAAGTTCTACTAC - 3´
CCL4<br>Stop codon insertion	Fwd 5´- CACC**TGGTGTGAGTATCAACCCCT** - 3´<br>Rev 5´- AAAC**AGGGGTTGATACTCACACCA** -3´
CCR1<br>Stop codon insertion	Fwd 5´- CACC**CACTCTCGTAGGCTTTCGTG** - 3´<br>Rev 5´- AAAC**CACGAAAGCCTACGAGAGTG** -3´
hIL-10<br>Stop codon insertion	Fwd 5´- CACC**GTTGTTAAAGGAGTCCTTGC** – 3´<br>Rev 5´- AAAC**GCAAGGACTCCTTTAACAAC** -3´
Neg ctrl sgDNA<br>Stop codon insertion	Fwd 5´- CACC**AAAGACTTGCTCCAAAACAC** - 3´<br>Rev 5´- AAAC**GTGTTTTGGAGCAAGTCTTT**-3´
Sequencing primer for gene deletion	5´- TACGATACAAGGCTGTTAGAGAG - 3´
B828 cloning	5’ACTGCCCCACTGTGCAATGCAGCTTTT<br>AGCCATGCCATGCTCAACAGCTATGACCATGATTACGCC-3’
B828 cloning	5’GTGAAATGCACCCATCTCCTGCTTCCA<br>GGGTATGGAATTACCAGTCACGACGTTGTAAAACGAC-3’
B738 cloning	5’GTCATAGTAG CTTAGCTGAA CTGGGCCGTG<br>GGGGTCGTCA AACAGCTATG ACCATGATTA CGCC-3’
B738 cloning	5’TACATAAGCC TCTGTCACTG CTCTGTCAGC<br>TTCTTTCCTC CCAGTCACGA CGTTGTAAAA CGAC-3’

Suppl. Tab. 1 Primers used for gene deletion (in bold the gDNA sequence) or cloning of an EBV mutant. The underlined external segments of teh primers are homologous to the BCRF1 gene.

Gene ID	***Fold change***	***Adjusted p value***
MALAT1	1.76	7.93E-146
B2M	1.55	6.23E-106
SNORD10	2.24	4.01E-102
TMSB4X	1.75	6.69E-102
RPS27	1.59	9.71E-96
RPL41	1.60	2.14E-89
MT-CO2	1.67	9.08E-88
MT-RNR1	1.28	1.74E-79
**CD52**	**2.18**	**2.42E-75**
MTATP6P1	1.77	3.39E-73
EEF2	-1.78	5.49E-71
MT-RNR2	1.24	4.62E-68
MT-CO3	1.51	3.14E-61
RPS28	1.58	4.16E-59
RPS29	1.51	1.78E-55
RPS19	1.40	1.04E-53
TMSB10	1.65	2.69E-52
MT-ATP6	1.53	4.50E-51
RPS18	1.55	7.90E-51
ENSG00000281383	1.33	8.45E-50

Suppl. Tab. 2 Top 20 regulated genes single cell RNAseq. The differential expression analysis was performed using the Wilcoxon Rank Sum test (two-sided test). P-values were adjusted using the Bonferroni correction.

Supplementary Note 1

Directionality

Directionality of a given path is defined as the ratio between the minimal Euclidian distance between the start point and the end point of the path (straight line joining them), divided by the total length of the cell track (accumulated distance). The global directionality of a migrating cell population is given as the mean of the directionalities of each individual cell track.

Mean displacement plot[1].

Information about the nature of the cell paths (random versus directed) can be obtained by plotting the mean displacement (shortest path between two recorded points) of migrating cells against the square root of time to generate a square root profile of these paths. If displacement is a linear function of the square root of time (graph forming a straight line), the movement is random Brownian. However, if the cell moves larger distances per time unit, the function grows more quickly than in a linear relation (graph above a straight line, sub-linear or hyperbolic), and the migration is likely directionally persistent.

Random walk model[2,3].

We simulated a purely "random walk", in which every time unit, each cell will move in a perfectly random direction at a random speed, independently of any previous movements of the same cell. Starting points were chosen randomly (uniform distribution) on a two-dimensional experimental area, with directions taken from a uniform distribution between 0 and 360°. Migration velocities were sampled from the empirical distribution of the actually observed velocities in the time lapse experiment (Suppl. Fig. 12).

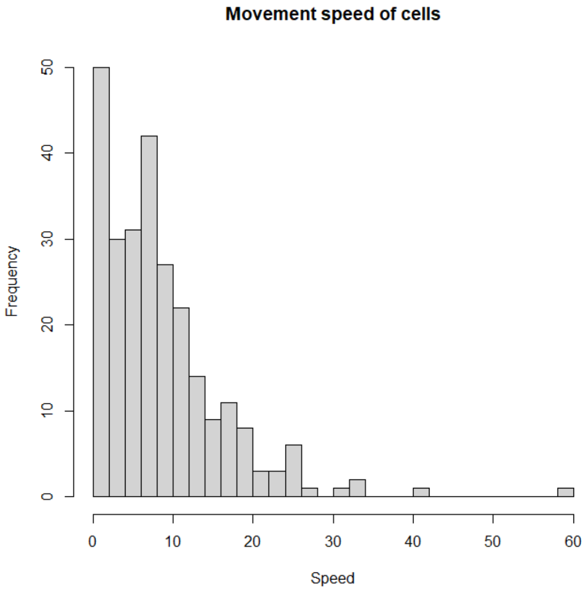

Suppl. Fig. 12: Distribution of empirical movement speeds per time unit, primary B cells stimulated with CD40L, IL-4 and CXCL12.

We then simulated a correlated random walk by determining the movements of a cell at any time point after the second as a weighted mean between the movement at the previous time point, and a new, randomly chosen movement[3]. The initial movement at $t = 1$ is considered to be completely random, as in the random walk. A weight variable w (chosen between 0 and 1) determines the strength of the correlation, and, by definition, a weight of $w = 0$ indicates a purely random walk, while a weight of $w = 1$ indicates a movement along a straight line. As the weight determines the distance covered by the cells (defined as distance from starting position to ending position), we can distinguish between purely and correlated random walks by simulating an empirical distribution of covered distances under $w = 0$ (i.e. a purely random walk), and comparing the actual distance to this distribution. Precisely, we simulated a random walk for a set of n cells over k time units, and calculate the average distance between starting and ending point each time. The data generating process was repeated a large number of times, in this case 1000 times. If the actually observed average distance for all cells is larger than the distance observed in 95% of the simulation runs, the null hypothesis of a purely random walk

(w = 0) can be rejected at an alpha = 5% level. The quantile position of the actual data in the distribution of the simulated runs provides the p-value.

Furthermore, simulating the average covered distances for different strengths of correlation w, and comparing these to the actually observed average distance provides an estimate for the strength of the correlation, that is, for the degree of directionality present in the real data set.

Suppl. Fig. 13 shows a correlated random walk with w=0.3 and w=1.0. The latter is a complete continuation of initial movements, resulting in straight line movements.

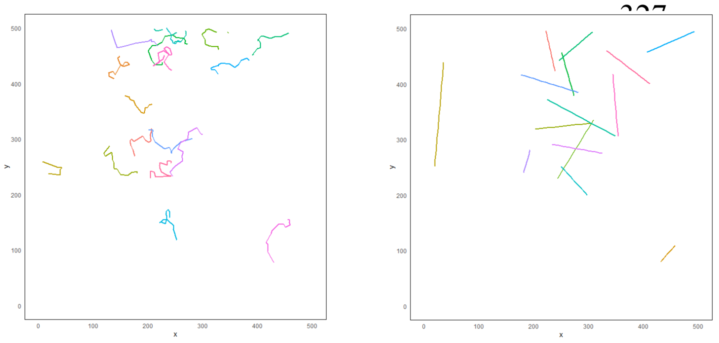

Suppl. Fig. 13: Correlated random walks with low (w=0.3, left) and complete (w=1.0, right) correlations, based on movement speeds from CXCL12 cells.

<u>Software:</u> All simulations were performed using R Statistical Software (v4.3.0; R Core Team 2023).

Supplementary References

1 Beltman, J. B., Maree, A. F. & de Boer, R. J. Analysing immune cell migration. *Nat Rev Immunol* **9**, 789-798 (2009). https://doi.org/10.1038/nri2638

2 Beauchemin, C., Dixit, N. M. & Perelson, A. S. Characterizing T cell movement within lymph nodes in the absence of antigen. *J Immunol* **178**, 5505-5512 (2007). https://doi.org/10.4049/jimmunol.178.9.5505

3 Renshaw, E. & Henderson, R. The Correlated Random-Walk. *J Appl Probab* **18**, 403-414 (1981). https://doi.org/Doi 10.2307/3213286
