# Supplementary material for: Epstein–Barr virus induces aberrant B cell migration and diapedesis via FAK-dependent chemotaxis pathways
Source: Nat Commun. 2025 May 19;16:4581. doi: 10.1038/s41467-025-59813-z (PMC12089463; doi:10.1038/s41467-025-59813-z)
Supplement: Supplementary file 2 — Description of Additional Supplementary Files [file 41467_2025_59813_MOESM2_ESM.pdf]

Description of Additional Supplementary Files

File name: Supplementary Movie 1
Description: Movement of EBV-infected B cells in a collagen matrix.
B cells infected with the Epstein-Barr virus were embedded in collagen at high density (3x10E5/ml) and observed for 15 minutes using time lapse microscopy.

File name: Supplementary Movie 2
Description: Movement of CD40L/IL-4/CXCL12 stimulated B cells in a collagen matrix.
B cells stimulated with CD40L, IL-4 and CXCL12 were embedded in collagen and observed for 15 minutes using time lapse microscopy.

File name: Supplementary Movie 3
Description: Movement of resting B cells in a collagen matrix.
Primary resting B cells were embedded in collagen and observed for 15 minutes using time lapse microscopy.

File name: Supplementary Movie 4
Description: Movement of DC40L/IL-4 stimulated B cells in a collagen matrix.
B cells stimulated with CD40L and IL-4 were embedded in collagen and observed for 15 minutes using time lapse microscopy.

File name: Supplementary Movie 5
Description: Movement of EBV-infected B cells in a collagen matrix at a low density.
B cells infected with the Epstein-Barr virus were embedded in collagen at low density (3x10E4/ml) and observed for 15 minutes using time lapse microscopy.

File name: Supplementary Movie 6
Description: Movement of defactinib treated EBV-infected B cells in a collagen matrix.
B cells infected with the Epstein-Barr virus were exposed to defactinib for 30 minutes (3.5 µM), embedded in collagen at high density (3x10E5/ml), and observed for 15 minutes using time lapse microscopy.

File name: Supplementary Movie 7
Description: Adhesion of EBV-infected B cells to BMEC.
The adhesion of EBV-infected B cells to a layer of HBMEC was investigated under constant flow conditions. An infected B cell rolling on the endothelium is shown.
